# A Few Close Friends? Adolescent Friendships’ Effect on Internalizing Symptoms Is Serially Mediated by Desire for More Friends and Social Goal Orientation

**DOI:** 10.1007/s10964-023-01780-z

**Published:** 2023-04-30

**Authors:** Reubs J. Walsh, Nikki C. Lee, Imke L. J. Lemmers-Jansen, Miriam Hollarek, Hester Sijtsma, Mariët van Buuren, Lydia Krabbendam

**Affiliations:** 1 Department of Clinical, Neuro-, and Developmental Psychology, Faculty of Behavior and Movement Sciences, Vrije Universiteit Amsterdam, Amsterdam, the Netherlands; 2 Institute for Brain and Behavior Amsterdam, Vrije Universiteit Amsterdam, Amsterdam, the Netherlands; 3 LEARN! Interfaculty Research Institute, Vrije Universiteit Amsterdam, Amsterdam, the Netherlands; 4 Department of Developmental Psychology, Utrecht University, Utrecht, the Netherlands; 5 Department of Psychosis Studies, Institute of Psychiatry, Psychology and Neuroscience, King’s College London, London, UK

**Keywords:** Early adolescence, Social cognition, Classmate friendships, Social goals, Motivation, Adjustment

## Abstract

Interpersonal connection is a fundamental human motivation, and the extent to which it is fulfilled is a strong predictor of symptoms of internalizing disorders such as social anxiety and depression, perhaps especially during the “social reorienting” period of adolescence. However, little is known about the contribution to this effect of the individual’s social motivations, which are intensified during adolescence. Furthermore, social goal orientation – an individual’s priorities and intentions in social interactions – is an important predictor of vulnerability to internalizing symptoms. Adolescents spend most of their waking lives in classrooms, bounded social networks with a limited pool of candidates for befriending. This study investigated whether friendships within one’s class protects against internalizing symptoms in part by reducing the desire for more classmate friendships, which may tend to promote maladaptive social goals. Participants were 423 young adolescents (*M* age = 13.2, *sd* = 0.52 years; 49.4% girls). As predicted, adolescents’ number of reciprocated classroom friendships had a protective effect on internalizing symptoms which was serially mediated by desire for more such friendships, and social goal orientation. However, only demonstration-avoidance goals significantly predicted internalizing symptoms. Unreciprocated friendship nominations were unexpectedly associated with stronger desire and more social anxiety symptoms. The results suggest that the effect of number of friends is mediated by the individual’s thoughts and feelings about their number of friendships, such that a strong desire for more friendships promotes maladaptive goals, oriented toward social status and consequently less oriented toward the cultivation of interpersonal intimacy with the friends they already have.

## Introduction

Adolescence is a sensitive window for the development of the motivation and capacity to navigate social interactions outside of the family. As children enter adolescence, desire to feel included in social groups intensifies, and becomes increasingly oriented towards peer friendships in preference to family. Adolescents’ reward systems are highly attuned to social reward, while their executive functions and social cognition continue gradually to mature (Crone & Dahl, 2012). These changes help motivate and equip the adolescent to complete the developmental tasks of adolescence, such as learning to establish and maintain interdependent peer relationships; becoming independent from family; the construction of a stable and autonomous social identity; and navigating varied social norms and expectations to manage others’ perception of them (Manning, 2002). These developmental tasks are social in nature and therefore depend upon the impact of social experiences on the adolescent’s cognitions and motivations. However, these effects are not always adaptive, and social experiences during this sensitive period dramatically influence individual vulnerability to internalizing symptoms (Rapee et al., 2019), into adulthood (Narr et al., 2019). Indeed, the social support of friendships is a powerful determinant of mental and physical health across the lifespan (Holt-Lunstad et al., 2015), but may be especially important in adolescence (Sebastian et al., 2010), both to facilitate psychosocial development, and because adolescents rejected by their peers may nevertheless withdraw from familial social support, leading to social isolation (Thomas & Bowker, 2015). However, it remains unknown how and how much adolescents’ goals and desires about their social interactions and circumstances contribute to the effect of friendships on internalizing. Additionally, for most adolescents, the majority of their social interactions will take place within the school environment. Many schools are highly structured, constraining which peers an individual may interact with, especially when individuals are assigned to a single class for most or all lessons. This amplifies the importance of classroom friendships to the adolescent. Despite this, the role of the motivations guiding an adolescent’s social cognition and behavior in the association between their classroom friendships and their risk of developing internalizing symptoms, is not yet well understood.

Although many aspects of adolescent friendship experiences have been studied as predictors of mental health outcomes, relatively little is known about the role of social motivation in these associations. Existing evidence suggests that adolescents’ social goals during this sensitive period markedly influence their vulnerability to internalizing symptoms (Kuroda & Sakurai, 2011; Ryan & Shim, 2008). It has been suggested that affect-laden, psychological needs-driven motives or desires regulate the selection and prioritization of goals to pursue (Thrash & Elliot, 2005). Therefore, desire for more friends may influence social goals such as acquiring social skills or status. If so, the effect of number of friendships on internalizing symptoms may arise partly or only because it predicts the extent to which a psychological need for peer-affiliation is satisfied, and so the strength of the desire for more friends. This may in turn drive social goal-pursuit behaviors and cognitions toward core features of internalizing, such as social withdrawal and/or hypervigilance to negative evaluation. Nevertheless, it remains unknown whether and how classroom friendships influence these goals, nor whether such influence contributes to the effect of friendships on the risk of internalizing symptoms.

### Adolescent Friendships and Internalizing Symptoms

The number of friends an adolescent has is a particularly powerful predictor of internalizing risk. Adolescents with no friends in their class have high rates of internalizing symptoms and tend to perceive school as socially threatening (Lessard & Juvonen, 2018), and for most adolescents, more reciprocated friendships is associated with fewer internalizing symptoms (Ueno, 2005). However, the mechanism by which a higher number of friends reduces the emergence of internalizing symptoms is unlikely to be (only) a direct effect, and indeed there is substantial evidence that the effect is partially mediated by a variety of constructs, including friendship quality, loneliness, and belonging (Lodder et al., 2017). Moreover, the effect is nonlinear, so that for adolescents with the very highest numbers of friendship ties, additional friendships do not reliably provide additional benefit; on the contrary, such adolescents tend to be more depressed (Falci & McNeely, 2009; c.f. Pachucki et al., 2015) and less socially content (Ferguson & Ryan, 2019) than those with a more moderate number. Nevertheless, the effect of adolescent friendship on internalizing symptoms has many possible mechanisms including those arising from the qualitative attributes of the friendships themselves, (Waldrip et al., 2008) and others arising from friends’ tendency to become more similar over time (Veed et al., 2019), for example.

Number of friends is especially important in the context of the “bounded community” of a classroom. While in unstructured settings, new candidates for friendship may be encountered relatively frequently, in the classroom network, the set of candidates is relatively fixed across time, and it is more difficult to avoid contact with particular individuals. Consequently, adolescents in such classes necessarily have a relationship with all (or most) of their classmates, including those who are not their friend, and it is in the context of this extant non-friend relationship that attempts to initiate friendship would take place. Under these circumstances, the desire for more friends may make non-friend relationships, and, by extension, absolute number of friends, more salient. Meanwhile, perceptions of one’s social competence and the social support available may be calibrated by these classroom experiences. Quantity of reciprocated friendships in the classroom is therefore an important predictor of adolescents’ vulnerability to internalizing symptoms, and its drivers are not necessarily limited to effects occurring at the dyadic level, such as social support, but also include effects at the intra-individual and whole-group (classroom) levels.

### Social Goals

Goal orientation (Dweck & Leggett, 1988) was originally developed to study differences in academic motivation and achievement (Elliott & Dweck, 1988), and has since been extended to the social domain (Ryan & Shim, 2006). In both cases, a distinction is drawn between an intrinsic desire to learn (academic “mastery” or social “development” goals) and the extrinsic desire to seem competent (“performance” or “demonstration” goals). Subsequently, demonstration (or performance) goals have been further divided into avoidant and approach dimensions, revealing that the association of demonstration goals with worse outcomes is driven primarily by the avoidant dimension, while demonstration-approach goals may even be beneficial in some cases, in both academic (Elliot & Harackiewicz, 1996) and social (Ryan & Shim, 2006, 2008) domains. However, these beneficial effects are conditional on development (mastery) goals also being endorsed, and on sociodemographic variables (Midgley et al., 2001). This highlights the fact that, at least at the level of goals, these factors are orthogonal, rather than opposing ends of a spectrum or discrete, mutually-exclusive states (Skaalvik, 1997).

Social Goal Orientation (SGO) can be measured with the Social Goals questionnaire (SGQ; Ryan & Shim, 2006, 2008), which asks adolescents about their priorities and intentions in their social interactions and has a three-factor structure. These factors measure (1) intrinsic goals seeking to learn new social skills and deepen existing friendships (“Development”); (2) extrinsic goals seeking to demonstrate social competence and/or (gain) status (“Demonstration-Approach”); and (3) extrinsic goals seeking to avoid demonstrating a lack of social competence and/or (losing) social status (“Demonstration-Avoidance”). The three SGQ subscales correlate differently with psychosocial adjustment variables. Development goals are associated with better adjustment across a range of variables including friendship satisfaction and loneliness (Liem, 2016), and self-acceptance and social worry (Shim et al., 2013). Demonstration-avoidance goals are associated with worse adjustment across variables including loneliness (Mouratidis & Sideridis, 2009), social worry (Shim et al., 2013), depression (Kuroda & Sakurai, 2011), and social anxiety (Shim & Ryan, 2012). Findings regarding demonstration-approach goals vary, similarly to academic performance-approach goals. While some studies found no association with social adjustment variables when the other two SGQ subscales were included in the model (e.g., Ryan & Shim, 2006, 2008), others found that demonstration-approach goals predicted greater belonging, but also greater loneliness, and feeling less accepted by peers (Mouratidis & Sideridis, 2009). The SGQ subscales are often related to closeness-seeking (development subscale), status-seeking (demonstration-approach) and status-preserving (demonstration-avoidance) (e.g., Rodkin et al., 2013; Rudolph et al., 2011; Ryan & Shim, 2008). In light of the associations of SGQ subscales with internalizing symptoms, this characterization is consistent with evidence that adolescents who achieve a status-oriented goal of greater popularity are at greater risk of internalizing symptoms, and that adolescents who achieve a closeness-oriented goal of increased friendship intimacy are at lower risk (Narr et al., 2019).

A previous study found that young adolescent girls with an unfulfilled desire to become friends with one or more peers were lonelier than those who did not, even after controlling for other social network measures (Thomas & Bowker, 2013). This suggests that adolescents’ experience of their social network position can be shaped by their desire to maintain or change that position. Nevertheless, it remains largely unknown what role inter-individual differences in adolescents’ desire to increase their number of classroom friendships plays in these social influences on internalizing symptoms (Rueger et al., 2016). On this basis, one may hypothesize that adolescents with fewer friends in their class are more vulnerable to internalizing symptoms in part because, within the bounded community of the classroom, a desire for more friendships may promote demonstration-oriented social (status) goals. Moreover, acquiring more friends in one’s class requires attending to relationships within the class that are not (yet) friendships. In this context, adolescents’ attention to non-friends may increase their use of metaperception (i.e., perceiving how one is perceived by others) in guiding behavior, as they seek to influence how their peers perceive them.

While adolescents with fewer friends will tend to have a stronger desire for more, an adolescent who strongly desires more friendships would be more vulnerable to internalizing symptoms than an adolescent with the same number of friends in their class, but a milder desire to increase that number. Such a desire could amplify the salience of non-friend relationships, motivating attempts to identify and conceal or alter (likely benign) behaviors or traits (Simone et al., 2018) that the adolescent perceives as hindering them in seeking friendship with those peers – consistent with demonstration-avoidance goals. Meanwhile, an adolescent with a less strong desire for more friends in their class would be less vulnerable to this mechanism, even if they have relatively few classroom friendships. Note, though that they would remain vulnerable to other mechanisms, such as a more limited pool of social support available to draw upon (Aune et al., 2020), amplified effects of conflict in those friendships they do have (Boersma-van Dam et al., 2019), and a diminished sense of belonging (Ueno, 2005).

If this is the case, it may be possible to intervene to reduce this contribution to adolescents’ internalizing risk. A simple, one-off, 30-minute psychoeducational intervention has successfully been used to shift adolescents’ beliefs about personality toward those (personalities can change, i.e., incremental, not entity, beliefs) which promote adaptive goals associated with less risk of internalizing – this intervention improved recovery after a social stressor (Schleider & Weisz, 2016). This has been successfully extended to beliefs about intelligence and self-control (Schleider et al., 2019), and to safeguard against potential side-effects, such as increased self-blame (Perkins et al. (2021)). A further extension of this intervention, also targeting beliefs about social competence and peer relationships (Rudolph, 2010) thought to shape SGO, could further reduce internalizing risk in adolescents (especially those with few friends) by guiding them toward seeking to develop their friendships and social skills by pursuing closeness, instead of seeking to express, obtain and retain social status. This kind of intervention could furthermore produce cultural change within schools. However, there may be changes to the structure of individual schools or whole education systems, that could establish and maintain a culture of social inclusion, in which benign behaviors and traits need not be concealed, non-friend relationships are more amicable, with friendship being defined more by closeness than by alliances and cliques, and peer victimization and exclusion behaviors less routinely rewarded with gains in social status (consider, e.g., Juvonen et al., 2019; Razer et al., 2013).

## The Present Study

The number of friends an adolescent has in their school environment is a strong predictor of their risk of developing internalizing symptoms and disorders (Sebastian et al., 2010), and some recent evidence suggests that SGO may also play an important role (e.g., Rodkin et al., 2013). How and to what extent these effects are connected remains unknown, but the theoretical considerations outlined above suggest a desire to increase one’s number of friends would mediate an association between the current number of friends, and SGO. This study sought to shed light on this question by investigating the contribution of desire for more friends in class and social goals to the relationship between adolescents’ number of classroom friendships and internalizing symptoms, focusing on interactions within the internal states of the adolescent, and in particular, their desire to increase their number of friends in their class. Thus, an effect of a comparatively “objective” measure of the school social environment an adolescent experiences (number of friends), on internal motivational states, is hypothesized to depend upon the adolescent’s (subjectively experienced and reported) affective and motivational response to that “objective” number (desire to increase number of friends in their class). To test whether the negative association between number of classroom friendships and internalizing symptoms is partially mediated, in two steps, by the desire to increase one’s number of classroom friendships, and SGO, in a nonclinical community sample of Dutch adolescents in their first and second year of secondary education. The path model arises from two hypotheses. Firstly, that the stronger an adolescent’s desire for more classroom friendships, the more that adolescent would adopt social goals and behaviors that undermine the protective effect of their extant friendships. If this is the case, the intensity of an adolescent’s desire for more classroom friendships should mediate the effect of number of friendships on internalizing symptoms. Secondly, that social goals would be intensified by a strong desire for more friends, and mediate an association between desire and internalizing symptoms by prioritizing either status-seeking (demonstration goals) or closeness-seeking (development goals) at the expense of the other, leading to more internalizing symptoms in demonstration-oriented adolescents. These hypotheses lead to six predictions. First, number of reciprocated and unreciprocated classmate friendships will each be negatively associated with internalizing symptoms, and this effect will be greater for reciprocated friendships. Second, number of reciprocated and, to a lesser extent, unreciprocated classmate friendships will also each be negatively associated with desire for more classmate friendships. Third, desire will mediate the effect of number of friendships on internalizing symptoms and on SGO. Fourth, desire will be positively associated with both demonstration, and to a lesser extent, development SGQ subscales. Fifth, the effect of number of friendships on internalizing symptoms will be serially mediated by desire and SGO. Finally, the indirect effects of number of friendships on internalizing symptoms mediated by demonstration goals will be negative (i.e., more friends leads to less desire, leads to less demonstration goals, leads to less internalizing symptoms), whereas the indirect effect mediated by development goals will be net-positive due to inconsistent mediation (i.e. fewer friends leads to more desire, leads to more development goals, but more development goals leads to less internalizing symptoms, when controlling for the negative direct effect, and the negative indirect effect through desire, of number of friends).

## Methods

### Participants

Participants were early adolescents enrolled in the first or second year of high-school (*M* age = 13.2 years, *sd* = 0.52 years, range: 11.7–14.7 years) in the Netherlands. Participants were recruited through schools participating in the #SOCONNeCT project, in two longitudinal cohorts (see also e.g., Sijtsma et al., 2021). These participants were drawn from academically selective mainstream secondary schools; such schools comprise a total of ~40% of pupils nationally.

A total of 948 adolescents were recruited to the project, 728 of whom had completed, in a single session, all the measures used in the present analyses during their first or second year of high-school. Peer nominations were used to ascertain participants’ number of friends in their class. Incomplete data introduce noise because the participant cannot nominate or be nominated by non-participants in the peer-nomination questionnaire. Therefore, participants from classes with <70% participation were excluded from the current analyses. This threshold was selected because simulation studies (e.g., Smith & Moody, 2013) indicate that 70% participation is adequate for a reliable estimate of the true value of the sociometric variable. Participants in classes excluded due to low classmate participation (*N* = 305 in 29 classes) did not significantly differ from the included sample on the questionnaire variables used (Beck Youth Inventory Depression Scale (BYI-D; *t* = −1.624, *p* = 0.105, *d* = 0.132), Social Anxiety Scale for Adolescents (SAS-A; *t* = −0.949, *p* = 0.343, *d* = 0.075), SGQ Development (*t* = 0.550, *p* = 0.582, *d* = 0.044), SGQ Demonstration-Avoidance (*t* = 0.757, *p* = 0.450, *d* = 0.059), SGQ Demonstration-Approach (*t* = 1.599, *p* = 0.110, *d* = 0.122)).

A total of 423 participants (across 19 classes with a mean participation rate of 83.3%), 209 of whom were girls (49.4%), were included. Age did not differ by reported sex (*t* = −0.266, *p* = 0.791, *d* = 0.042).

### Measures

The Cronbach’s alpha values reported in this section were calculated for the present sample. The internalizing symptoms studied as outcome measures were those associated with social anxiety and depression, as these are closely associated with both inter- and intrapersonal social risk factors (such as low peer acceptance and negative self-image, respectively), and are relatively common in adolescent samples (Rapee et al., 2019).

#### Peer nominations

In response to nine typical sociometric nomination questions (Cillessen & Marks, 2017), participants nominated classmates by selecting their names from a list, presented in a grid on a tablet screen. The list was composed of all the names of participating classmates, and excluded those who did not consent to participate in the study. For the planned analyses, the responses to the “Who on this list are your friends (maximum 15)?” item were used; this was the only item for which the number of nominations was limited. The nomination lists include only participating classmates, so participation rates below 100% do not affect the ability to determine which friendship nominations are reciprocated, as participants were only able to nominate classmates who could potentially return the nomination. This allows one to distinguish a more “objective” measure of the participant’s friendships from a more “subjective” measure that reflects the participant’s perception of their friendships. References to “reciprocal” friendships will hereafter be used to mean (the number of) friendships that were reported by both the participant and the friend, and “unreciprocated” friendships, will be used to mean (the number of) friendship nominations by the participant where the nominated friend did not nominate the participant. The items “With whom on this list do you talk about your feelings?”, “Who on this list is popular?”, and “Who on this list is likable?” were used in the post-hoc analyses.

#### Social network perception

Participants were also asked to report their perception of their network and, in particular, to rate statements that they desired to 1. have more friends in their class, 2. be more popular with classmates, and 3. be liked more by classmates. Responses to each question were on a five-point scale between “not at all” and “very much so”. The response to the first item is referred to hereafter as “desire” (i.e., desire to have more friends). Due to the single-item nature of each of these measures, the resultant variables are treated as ordinal.

#### Social goals questionnaire (SGQ)

Ryan and Shim (2006) developed and validated a three-factor social achievement goals questionnaire in college students, which assesses the participant’s approach to social interactions based on an earlier study (Erdley et al., 1997). This questionnaire was adapted for use with children (Rudolph et al., 2011). A Dutch-language version of this questionnaire was administered to participants after in-house translation. Items with a factor-loading greater than 0.50 were translated by a natively bilingual speaker of Dutch and English and back-translated into English to confirm the accuracy of the translation. The three-factor structure comprises development (6 items, α = 0.86), demonstration-approach (5 items, α = 0.86) and demonstration-avoidance (7 items, α = 0.84) subscales. Responses are on a five-point scale between “never” (1) and “always” (5). Subscale scores were calculated as the mean of the responses on the items in that subscale, with higher scores indicating greater endorsement of the corresponding goal-orientation factor.

#### Social anxiety scale for adolescents (SAS-A)

This questionnaire evaluates the extent of social anxiety symptomatology in adolescents (for validation see; Inderbitzen-Nolan & Walters, 2010; La Greca & Lopez, 1998). A Dutch-language translation of SAS-A with consistently high Cronbach’s alpha was used (Blöte, et al., 2010; Blöte & Westenberg, 2007; Miers, et al., 2013). It is comprised of three factors: fear of negative evaluation (8 items, α = 0.92), novelty-related social avoidance and distress (6 items, α = 0.84) and general social avoidance and distress (4 items, α = 0.78). Responses to each item were on a five-point scale between “not at all” and “very much so”. Scores were calculated as the unweighted sum of the responses on all 18 items (α = 0.93), with higher scores indicating that the participant reports experiencing more social anxiety symptoms.

#### Beck youth inventory depression scale (BDI-Y)

The BDI-Y evaluates the extent of depressive symptomatology in children and adolescents aged 7–18 and has 20 items in a single factor (α = 0.93). A validated Dutch translation of this validated questionnaire was used (Beck et al., 2005). Responses are on a four-point scale between “never” and “always”. Scores were calculated as the unweighted sum of the responses on all 20 items, with higher scores indicating that the participant reports experiencing more depression symptoms.

### Procedure

Both the participant and their parent(s) or guardian(s) provided written informed consent to participate in the #SOCONNeCT project following a letter and an information evening to inform them of the protocol and aims of the study and their rights as participants. Participation took place under exam conditions in the classroom setting, supervised by members of the research team, and lasted approximately 90 min (plus breaks) including explanation and administration of all behavioral measures for the wave, including several which are not used in the present analysis. Throughout data collection participants were able to ask questions to the researchers. Each participant completed the questionnaires individually on an iPad provided by the researchers. All materials and the means of administration (i.e., iPads) were tested and validated in focus groups, which were also used to ensure that the information provided was sufficient and to compile prepared answers to frequent queries. Payment of €7.50 per participant was made to the school to be spent on a class activity. The #SOCONNeCT project was approved by the Scientific and Ethical Review Board of the Faculty of Behavioral and Movement Sciences of the Vrije Universiteit Amsterdam.

### Data Preprocessing

Missing data were handled by listwise deletion; peer nomination data from classes with below 70% participation were considered unreliable, and therefore treated as missing (i.e., all data from such classes were excluded from the analyses). Peer nomination data were corrected for effects of class size and participation rate using a method adapted from a previous study (Velásquez et al., 2013). This method mitigates the effect of the missing peer nomination data that results from less than 100% participation within a given class. Questionnaire and peer nomination data were then normalized and centered. Please see the supplementary materials for more details on each of these steps.

### Planned Path Analyses

**Fig. 1:**
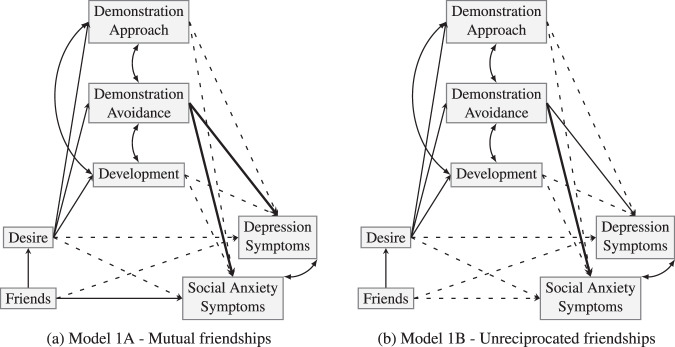
Models for (**a**) reciprocated and (**b**) unreciprocated friendships. Straight lines are freely-estimated regressions, curved lines are freely-estimated covariances. Regressions with an estimate <1 are bold lines, and those which failed to reach significance (i.e., *p* < 0.05) are dashed lines

Planned analyses for the present study consisted of two unnested path models to separately examine effects of reciprocated friendships (model 1A) and unreciprocated friendships (model 1B), as shown in Fig. 1. This analysis used *R* (R Core Team, 2020) and the *lavaan* package (Rosseel, 2012) in *RStudio* (RStudio Team (2020)). Means, errors and variances were freely estimated, as were the covariances of the three SGQ subscales, and the covariance of the SAS-A (*Social Anxiety*) and BDI-Y (*Depression*).

The following model-test statistics were used: Comparative Fit Index (CFI): values >0.94 indicate good model fit; Root Mean Square Error of Approximation (RMSEA): values <0.08 indicate acceptable, and <0.06 good fit of the residuals (Hu & Bentler, 1999). Throughout, RMSEA values are accompanied by a *p*-value representing the probability that the true value of RMSEA lies below 0.05. Values above *p* = 0.05 are typically interpreted as indicating that the RMSEA distribution does not recommend model rejection. Due to the use of a robust DWLS (diagonally-weighted least squares) estimation, information criteria cannot be calculated. In all cases, the DWLS-robust estimate of the fit statistic is reported. These planned analyses were followed up with univariate regressions examining the estimated regression paths individually; a linear regression examining the association of demonstration goals with desires to be popular and to be liked, as measures of participants’ desire for social status; and linear regressions examining the associations of desire for more friends, depression, and social anxiety (as the dependent variables) with numbers of reciprocated and unreciprocated friendship ties.

## Results

### Friendship Effects’ Path to Psychopathology

#### Planned models

Descriptive statistics and correlations of all path-model variables are given in Table 1. Both models converged normally, and are depicted in Fig. 1. Table 2 shows the parameter estimates for these models. Table 3 gives Sobel estimates of the total effect, indirect effects, and the direct effect, of number of (un/reciprocated) friendship nominations on social anxiety symptoms and on depressive symptoms, including the ratio of each (in)direct effect to the total effect. Model 1A (in which the corrected estimate of number of reciprocated friendship nominations was the “*friends*” variable), performed well (CFI = 0.993, RMSEA = 0.059 (0.314)). Model 1B (in which the corrected estimate of number of unreciprocated friendship nominations was the “Friends” variable) performed less well (CFI = 0.987, RMSEA = 0.084 (0.130)). Non-nested models cannot be formally compared. In both cases, the effect of friends on depression and social anxiety was mediated in two steps; friends predicted desire, which predicted SGQ subscale scores, one of which (demonstration-avoidance) significantly predicted social anxiety and depression symptoms. This effect amounted to between ~18 and 53% of the total effect of number of (un/reciprocated) friendship nominations on social anxiety and depression symptoms. All other indirect effects were nonsignificant. The total effects of reciprocated friendships on social anxiety and depression symptoms were significant, as was the total effect of unreciprocated friendships on social anxiety symptoms. However, the total effect of unreciprocated friendship nominations on depression symptoms was nonsignificant. Moreover, the direct effects of reciprocated friendship nominations on depression symptoms, and of unreciprocated friendship nominations on social anxiety symptoms and on depression symptoms, were all nonsignificant, while the direct effect of reciprocated friendship nominations on social anxiety symptoms was significant, amounting to ~61% of the total effect.

**Table 1:** Means, standard deviations, and correlations for all model variables

	Mean	SD	1.	2.	3.	4.	5.	6.	7.	8.
1. Desire	2.118	1.162		3.622***	8.427***	3.592***	8.296***	7.134***	−6.662***	3.866***
2. Depression Symptoms	7.953	7.956	3.860***		11.911***	1.658	4.630***	2.859**	−2.307*	1.385
3. Social Anxiety Symptoms	38.612	11.81	8.438***	12.327***		4.124***	14.759***	6.523***	−4.999***	2.341*
4. Development	3.148	0.850	3.701***	2.247*	4.803***		6.747***	3.179**	−0.149	2.718**
5. Demonstration-Avoidance	2.990	0.810	8.033***	4.947***	15.154***	7.000***		9.162***	−3.977***	1.373
6. Demonstration-Approach	2.194	0.809	7.167***	3.133**	7.207***	3.635***	9.420***		−0.651	2.779**
7. Reciprocated Friendships	4.111	2.201	−7.194***	−2.202*	−5.356***	−0.074	−3.945***	−0.721		1.468
8. Unreciprocated Friendships	2.466	2.196	3.859***	1.643	2.114*	2.722**	1.335	2.717**	1.462	

Correlations between the untransformed variables are given above the diagonal, and their correlations after the square-root transformation are given below the diagonal

*p ≤ 0.05; **p ≤ 0.01; ***p ≤ 0.001

**Table 2:** Regression weights and covariance estimates per path for Models 1A & B

Path			Model 1A		Model 1 B	
			Estimate (SE)	CSS Estimate (SE)	Estimate (SE)	CSS Estimate (SE)
Friends	→	Desire	−0.581 (0.069)***	−0.348 (0.042)***	0.358 (0.084)***	0.198 (0.047)***
Friends	→	Social Anxiety	−0.240 (0.074)**	−0.156 (0.049)**	−0.049 (0.077)	−0.027 (0.042)
Friends	→	Depression	−0.138 (0.119)	−0.057 (0.049)	0.046 (0.129)	0.017 (0.048)
Desire	→	Social Anxiety	0.087 (0.052)	0.094 (0.055)	0.360 (0.202)	0.358 (0.179)*
Desire	→	Depression	0.101 (0.090)	0.070 (0.062)	0.318 (0.204)	0.215 (0.136)
Desire	→	Development	0.037 (0.018)*	0.149 (0.070)*	0.088 (0.030)**	0.344 (0.109)**
Desire	→	Demonstration-Approach	0.073 (0.025)**	0.267 (0.089)**	0.159 (0.050)**	0.547 (0.146)***
Desire	→	Demonstration-Avoidance	0.082 (0.027)**	0.339 (0.103)***	0.152 (0.048)**	0.581 (0.153)***
Demonstration-Approach	→	Social Anxiety	0.250 (0.133)	0.074 (0.039)	0.029 (0.264)	0.008 (0.076)
Demonstration-Approach	→	Depression	0.245 (0.263)	0.046 (0.049)	0.046 (0.351)	0.009 (0.069)
Demonstration-Avoidance	→	Social Anxiety	1.889 (0.161)***	0.494 (0.039)***	1.685 (0.323)***	0.439 (0.086)***
Demonstration-Avoidance	→	Depression	1.001 (0.320)**	0.167 (0.053)**	0.821 (0.394)*	0.145 (0.067)*
Development	→	Social Anxiety	0.109 (0.151)	0.029 (0.041)	−0.002 (0.188)	0.000 (0.048)
Development	→	Depression	0.174 (0.298)	0.030 (0.052)	0.073 (0.323)	0.013 (0.056)
Development	↔	Demonstration-Approach	0.009 (0.003)*	0.132 (0.051)**	0.003 (0.005)	0.053 (0.087)
Development	↔	Demonstration-Avoid	0.016 (0.003)***	0.284 (0.046)***	0.011 (0.005)*	0.221 (0.074)**
Demonstration -Approach	↔	Demonstration-Avoid	0.020 (0.004)***	0.344 (0.048)***	0.013 (0.008)	0.246 (0.119)*
Depression	↔	Social Anxiety	0.472 (0.053)***	0.469 (0.036)***	0.452 (0.067)***	0.459 (0.044)***

→ represents regressions and ↔ represents covariances

*SE* standard error; *CSS* completely standardized solution

*p ≤ 0.05; **p ≤ 0.01; ***p ≤ 0.001

#### Respecified models

**Fig. 2:**
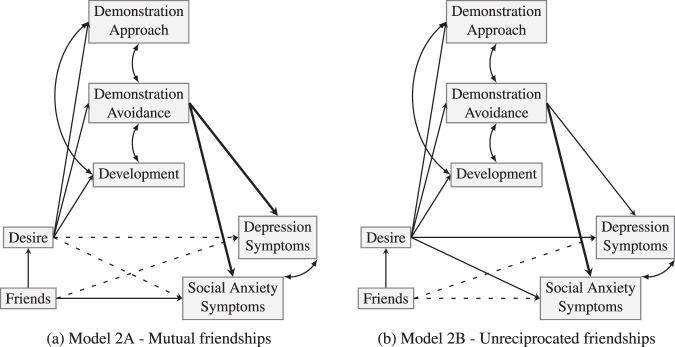
Respecified models for (**a**) reciprocated and (**b**) unreciprocated friendships. Straight lines are freely- estimated regressions, curved lines are freely-estimated covariances. Regressions with an estimate >1 are bold lines, and those which failed to reach significance (i.e., *p* > 0.05) are dashed lines

The presence of very small, statistically-insignificant regression estimates for several paths in Models 1A and 1B suggested the need to respecify the model. In particular, only one SGQ subscale– *demonstration-avoidance* – appeared to be predictive of internalizing symptoms. All regression weights for paths with the other two subscales as the predictor were consequently fixed to 0, and the resultant models 2A and 2B (see Fig. 2) were compared with models 1A and 1B, respectively. Both models improved (A: ∆χ^2^ = 0.467, (*p* = 0.977), CFI > 0.999, RMSEA < 0.008 (0.855); B: ∆χ^2^ = 3.296, (*p* = 0.510), CFI = 0.987, RMSEA = 0.050 (0.445)), and are depicted in Fig. 2. Table 4 shows the parameter estimates for these models. Table 5 gives Sobel estimates of the total effect, indirect effects, and the direct effect, of number of (un/reciprocated) friendship nominations on social anxiety symptoms and on depressive symptoms, including the ratio of each (in)direct effect to the total effect. The two-step mediations observed in models 1A and 1B were also observed in models 2A and 2B, amounting to between ~22 and 53% of the total effect. In addition, as in models 1A and 1B, the total effect of unreciprocated friendship nominations on depression symptoms was the only instance of a nonsignificant total effect in these models, and the direct effect of reciprocated friendship nominations on social anxiety was the only instance of a significant direct effect in these models, amounting to ~59% of the total effect. In addition, as in models 1A and 1B, the total effects of reciprocated friendship nominations on depression and social anxiety symptoms were significant, as was the total effect of unreciprocated friendship nominations on social anxiety symptoms. However, as in model 1B, the total effect of unreciprocated friendship nominations on depression symptoms was nonsignificant. Also as in models 1A and 1B, the direct effects of reciprocated friendship nominations on depression symptoms, and of unreciprocated friendship nominations on social anxiety symptoms and on depression symptoms, were all nonsignificant, while the direct effect of reciprocated friendship nominations on social anxiety symptoms was significant, amounting to ~59% of the total effect. Unlike model 1B, however, model 2B revealed significant one-step mediation effects of unreciprocated friendship nominations on social anxiety and depression symptoms, mediated by desire, amounting to ~77% and ~59% of the total effects, respectively.

**Table 3:** Indirect, direct and total effect estimates (sobel) of friends on internalizing for Models 1A & B

DV	Effect (Mediators)	Model 1A			Model 1B		
		Estimate (SE)	CSS Estimate (SE)	Ratio	Estimate (SE)	CSS Estimate (SE)	Ratio
Social Anxiety	Indirect (Desire)	−0.050 (0.031)	−0.033 (0.020)	0.128	0.129 (0.071)	0.071 (0.035)*	0.745
	Indirect (Desire, Demonstration-Avoidance)	−0.090 (0.032)**	−0.058 (0.020)**	0.228	0.092 (0.024)***	0.050 (0.012)***	0.530
	Indirect (Desire, Demonstration-Approach)	−0.011 (0.007)	−0.007 (0.004)	0.027	0.002 (0.015)	0.001 (0.008)	0.010
	Indirect (Desire, Development)	−0.002 (0.003)	−0.002 (0.002)	0.006	0.000 (0.006)	0.000 (0.003)	0.000
	Direct	−0.240 (0.074)**	−0.156 (0.049**	0.611	–0.049 (0.077)	−0.027 (0.042)	−0.284
	Total Effect	−0.393 (0.071)***	−0.255 (0.043***		0.173 (0.079)*	0.095 (0.042)*	
Depression	Indirect (Desire)	−0.059 (0.053)	−0.024 (0.022)	0.226	0.114 (0.072)	0.043 (0.026)	0.543
	Indirect (Desire, Demonstration-Avoidance)	−0.048 (0.022)*	−0.020 (0.009)*	0.184	0.045 (0.021)*	0.017 (0.008)*	0.213
	Indirect (Desire, Demonstration-Approach)	−0.010 (0.012)	−0.004 (0.005)	0.040	0.003 (0.020)	0.001 (0.007)	0.012
	Indirect (Desire, Development)	−0.004 (0.007)	−0.002 (0.003)	0.015	0.002 (0.010)	0.001 (0.004)	0.011
	Direct	−0.138 (0.119)	−0.057 (0.049)	0.535	0.046 (0.129)	0.017 (0.048)	0.221
	Total Effect	−0.259 (0.108)*	−0.107 (0.044)*		0.210 (0.126)	0.078 (0.047)	

Ratio is the (in)direct effect divided by the total effect

*SE* standard error; *CSS* completely standardized solution

*p ≤ 0.05; **p ≤ 0.01; ***p ≤ 0.001

**Table 4:** Regression weights and covariance estimates per path for Models 2A & B

Path			Model 2A		Model 2B	
			Estimate (SE)	CSS Estimate (SE)	Estimate (SE)	CSS Estimate (SE)
Friends	→	Desire	−0.575 (0.069)***	−0.344 (0.043)***	0.354 (0.082)***	0.195 (0.046)***
Friends	→	Social Anxiety	−0.232 (0.075)**	−0.150 (0.049)**	−0.053 (0.070)	−0.029 (0.038)
Friends	→	Depression	−0.129 (0.120)	−0.054 (0.050)	0.041 (0.126)	0.015 (0.047)
Desire	→	Social Anxiety	0.099 (0.055)	0.107 (0.058)	0.379 (0.107)	0.374 (0.093)***
Desire	→	Depression	0.116 (0.092)	0.080 (0.063)	0.346 (0.126)**	0.234 (0.083)**
Desire	→	Development	0.040 (0.018)*	0.158 (0.070)*	0.091 (0.020)***	0.354 (0.071)***
Desire	→	Demonstration-Approach	0.077 (0.025)**	0.284 (0.089)**	0.164 (0.030)***	0.560 (0.085)***
Desire	→	Demonstration-Avoidance	0.085 (0.026)**	0.355 (0.102)***	0.156 (0.031)***	0.591 (0.097)***
Demonstration-Avoidance	→	Social Anxiety	2.159 (0.179)***	0.558 (0.036)***	1.672 (0.282)***	0.434 (0.073)***
Demonstration-Avoidance	→	Depression	1.281 (0.327)***	0.211 (0.052)***	0.840 (0.375)*	0.149 (0.065)*
Development	↔	Demonstration-Approach	0.008 (0.003)*	0.129 (0.051)*	0.003 (0.004)	0.048 (0.064)
Development	↔	Demonstration-Avoid	0.016 (0.003)***	0.297 (0.051)***	0.011 (0.004)**	0.218 (0.062)***
Demonstration-Approach	↔	Demonstration-Avoid	0.022 (0.004)***	0.377 (0.051)***	0.012 (0.006)*	0.242 (0.089)**
Depression	↔	Social Anxiety	0.453 (0.055)***	0.462 (0.038)***	0.446 (0.061)***	0.455 (0.041)***

→ represents regressions and ↔ represents covariances

*SE* standard error; *CSS* completely standardized solution

*p ≤ 0.05; **p ≤ 0.01; ***p ≤ 0.001

**Table 5:** Indirect, direct and total effect estimates (sobel) of friends on internalizing for Models 2A & B

DV	Effect (Mediators)	Model 2A			Model 2B		
		Estimate (SE)	CSS Estimate (SE)	Ratio	Estimate (SE)	CSS Estimate (SE)	Ratio
Social Anxiety	Indirect (Desire)	−0.057 (0.033)	−0.037 (0.021)	0.144	0.134 (0.047)**	0.073 (0.024)**	0.774
	Indirect (Desire, Demonstration-Avoidance)	−0.105 (0.035)**	−0.068 (0.021)**	0.267	0.092 (0.023)***	0.050 (0.012)***	0.532
	Direct	−0.232 (0.075)**	−0.150 (0.049)**	0.589	−0.053 (0.070)	−0.029 (0.038)	−0.306
	Total Effect	−0.393 (0.071)***	−0.255 (0.043)***		0.173 (0.079)*	0.094 (0.042)*	
Depression	Indirect (Desire)	−0.067 (0.054)	−0.028 (0.022)	0.259	0.122 (0.050)*	0.046 (0.019)*	0.584
	Indirect (Desire, Demonstration-Avoidance)	−0.062 (0.024)**	−0.026 (0.010)**	0.241	0.046 (0.021)*	0.017 (0.008)*	0.221
	Direct	−0.129 (0.120)	−0.054 (0.050)	0.500	0.041 (0.126)	0.015 (0.047)	0.195
	Total Effect	−0.259 (0.108)*	−0.107 (0.044)*		0.210 (0.126)	0.078 (0.047)	

Ratio is the (in)direct effect divided by the total effect

*SE* standard error; *CSS* completely standardized solution

*p ≤ 0.05; **p ≤ 0.005; ***p ≤ 0.001

### Post-hoc Analyses

These analyses used *R* (R Core Team, 2020) and the *lme4* (Bates et al., 2015) and *QuantPsyc* (Fletcher, 2012) packages in *RStudio* (RStudio Team (2020)).

#### Univariate regressions

To facilitate comparison with the model-based estimates, univariate analyses were run, corresponding to each freely-estimated regression (i.e., path) in any model. The results of these analyses are shown in Table 6. Note the inverted associations with other variables when comparing reciprocated and unreciprocated friendships.

**Table 6:** Univariate regressions of variables sharing a path in any model

Path			Estimate (ß)	t-statistic	R^2^	F-statistic
Reciprocated Friends	→	Desire	−0.331***	−7.194	0.109	51.75
Reciprocated Friends	→	Social Anxiety	−0.253***	−5.356	0.064	28.69
Reciprocated Friends	→	Depression	−0.107*	−2.202	0.011	4.847
Unreciprocated Friends	→	Desire	0.185***	3.859	0.034	14.89
Unreciprocated Friends	→	Social Anxiety	0.103*	2.114	0.011	4.471
Unreciprocated Friends	→	Depression	0.080	1.643	0.006	2.699
Desire	→	Social Anxiety	0.380***	8.438	0.145	71.20
Desire	→	Depression	0.185***	3.860	0.034	14.90
Desire	→	Development	0.178***	3.701	0.032	13.70
Desire	→	Demonstration-Approach	0.330***	7.167	0.109	51.36
Desire	→	Demonstration-Avoidance	0.365***	8.033	0.133	64.54
Demonstration-Approach	→	Social Anxiety	0.331***	7.207	0.110	51.94
Demonstration-Approach	→	Depression	0.151**	3.133	0.023	9.817
Demonstration-Avoidance	→	Social Anxiety	0.594***	15.15	0.353	229.6
Demonstration-Avoidance	→	Depression	0.234***	4.947	0.055	24.48
Development	→	Social Anxiety	0.228***	4.803	0.052	23.07
Development	→	Depression	0.109*	2.247	0.012	5.047

*p ≤ 0.05; **p ≤ 0.005; ***p ≤ 0.001

#### Demonstration as status-seeking

Demonstration goals overlap conceptually with status-seeking behaviors, and this overlap was an important factor in the formulation of the hypotheses investigated here. To test whether this relationship held for this sample, a linear regression with (total, α = 0.86) *demonstration goals* as the dependent variable, and self-report desire for *popularity* and to be *liked* as the predictors, was performed. This regression was statistically significant (F(2,420) = 105.1, *p* < 0.001, adjusted *R*^2^ = 0.330). The main effects of desire for *popularity* (*t* = 9.398, *p* < 0.001, β = 0.454), and desire to be *liked* (*t* = 3.793, *p* < 0.001, β = 0.183) on *demonstration goals* were also significant. Note that the coefficient of desire for *popularity* exceeds double that of desire to be *liked*. This is consistent with the idea that demonstration goals overlap with status-orientation.

#### Comparison of reciprocated and unreciprocated friendships

As mentioned above, associations between friendships and other variables were in opposite directions depending on reciprocity. It is not possible to formally compare models A and B because the underlying data for the friendship variable are different, and the models are consequently unnested. Therefore, to compare the contributions of the two different measures of friendship in the social network, the corrected estimates of reciprocated and unreciprocated friendships were entered as independent variables into linear regressions predicting the *desire*, *depression* and *social anxiety* variables.

The linear regression with *desire* as the dependent variable was statistically significant (F(2,416) = 35.25, *p* < 0.001, adjusted *R*^2^ = 0.141). The main effects of number of *reciprocated* friends (*t* = −7.509, *p* < 0.001, β = −0.342), and number of *unreciprocated* friends (*t* = 4.500, *p* < 0.001, β = 0.205) on desire were significant. Note that contrary to expectations (*P2b*), an increase in the number of reported *unreciprocated* friendships was associated with an increase in the desire for more friendships, while *reciprocated* friendships was associated with a decrease in *desire*.

The linear regression with *depression* as the dependent variable was statistically significant (F(2,416) = 3.571, *p* = 0.029, adjusted *R*^2^ = 0.012). The main effect of *reciprocated* friends (*t* = −2.324, *p* = 0.021, β = −0.116), was significant, but the effect of *unreciprocated* friends was not (*t* = 1.548, *p* = 0.122, β = 0.076). Participants with more *reciprocated* friendships reported fewer depressive symptoms, but any association that may exist with *unreciprocated* nominations was too small to detect here. It may still be worth noting, however, that the coefficient estimates for the *reciprocated* and *unreciprocated* terms in this regression were once again in opposite directions.

The linear regression with *social anxiety* as the dependent variable was statistically significant (F(2,416) = 17.17, *p* < 0.001, adjusted *R*^2^ = 0.072). The main effects of *reciprocated* friends (*t* = −599, *p* < 0.001, β = −0.265), and *unreciprocated* friends (*t* = 2.291, *p* = 0.023, β = 0.109) were significant. Note that once again the influence of *unreciprocated* friendships runs counter to that of *reciprocated* friendships. Fewer *reciprocated* friendship nominations was associated with higher levels of social anxiety but, contrary to prediction, fewer *unreciprocated* nominations was associated with lower levels of social anxiety.

## Discussion

Adolescence is characterized by a dramatic increase in sensitivity to social reward, which motivates adolescents to pursue social acceptance, especially from peers (Crone & Dahl, 2012). This motivation serves the developmental tasks of adolescence, which depend on learning from social experience (Manning, 2002). However, adolescents’ intense social motivation also contributes to their marked vulnerability to internalizing symptoms (Rapee et al., 2019). Moreover, the specific goals an adolescent’s social motivation drives them to pursue – their social goal orientation (SGO) – predict differences in vulnerability to internalizing symptoms (Ryan & Shim, 2008). Similarly, adolescents with larger numbers of friendships have lower rates of internalizing symptoms and disorders including social anxiety (Rapee et al., 2019) and depression (Rueger et al., 2016). This study investigated whether friendships within one’s class may influence internalizing symptoms in part by reducing the desire for more classmate friendships, which may promote maladaptive social goals. A pair of path analyses were performed to test the effects of number of friendships on internalizing symptoms, using (A) only reciprocated and (B) only unreciprocated friendships. The results of path analysis (A) provide evidence that the effect of the number of reciprocated friends on internalizing symptoms is serially mediated by the desire for more friends, and SGO; specifically, demonstration-avoidance goals.

In the path models (A), as predicted, an association between numbers of reciprocated friendships and internalizing symptoms was mediated in two steps; number of reciprocated friendships negatively predicted desire, which in turn predicted more demonstration-avoidance goals, which in turn predicted more symptoms of depression and social anxiety. This was the only path to significantly predict depressive symptoms, and the only indirect path to significantly predict social anxiety symptoms (a significant direct effect of number of friends on social anxiety symptoms remained after controlling for desire and SGO). This confirms the prediction that internalizing symptoms would be positively associated with demonstration goals, but the predicted negative association with development goals was not found. Nevertheless, that the data supported this model suggests that the association between number of friends and internalizing symptoms is indeed driven in part by a tendency for adolescents who strongly desire more friendships to pursue maladaptive social goals, perhaps by encouraging a tendency to evaluate one’s social success in terms of status. One mechanism that could contribute to this (re)orientation toward status is that, from the perspective of low-status adolescents, their high-status peers have more friendships, and the quality (and sincerity) of those friendships cannot be evaluated from outside. Thus, for those adolescents who feel that they have too few friends, their social status seems to be synonymous with social inclusion and the avoidance of social exclusion. This is consistent with the observation here that desire was positively associated with both the demonstration-approach and demonstration-avoid goals subscales, which have been associated with status-seeking and -preserving in several past studies (e.g., Rodkin et al., 2013; Rudolph et al., 2011; Ryan & Shim, 2008). Moreover, a post-hoc analysis showed that demonstration goals were tightly, positively associated with the desire to be popular, and less strongly associated with the desire to be liked, which supports the suggestion that demonstration goals reflect a pursuit of social status in preference over interpersonal connection and closeness. One might therefore expect that demonstration-avoidance (status-preserving) goals would predispose the adolescent to differences in information processing conceptually similar to those associated with social anxiety. By drawing their attention to the possibility that aspects of one’s social behavior could be evaluated negatively by peers (Vassilopoulos & Banerjee, 2008), status-preserving goals may promote attentional bias toward “negative self-related social information” (Mellings & Alden, 2000, p. 254), setting them on a developmental pathway towards social anxiety disorder.

The finding that demonstration-avoidance goals were the only goals subscale to significantly positively predict internalizing symptoms is congruent with the goal orientation literature, which shows the most consistent harmful effects are driven by demonstration-avoidance goals (e.g. Kuroda & Sakurai, 2011; Ryan & Shim, 2008), while demonstration-approach goals often have a mixture of beneficial and harmful effects, and are highly contingent on individuals’ traits and circumstances (e.g. Mouratidis & Sideridis, 2009). However, while development goals have generally been shown to have protective or ameliorative effects (e.g., Kuroda & Sakurai, 2011; Shim et al., 2013), the present results indicate a positive association with internalizing symptoms, which was statistically significant only at the univariate level. One possible interpretation of this is that development goals are more likely to be endorsed by individuals who feel that their social skills are lacking, and thus an unmodeled, positive path from social anxiety to development goals (i.e., in the opposite direction to the path included in the model) could have concealed a protective effect of development goals. Related to this, in the academic domain, a similar approach/avoidance division for development goals has been suggested, to distinguish the pursuit of increased skill from the avoidance of decreases in skill (Lou, Masuda & Li, 2017); if such a distinction is justified, the combination of both approach and avoidance within one SGQ subscale may have obscured positive effects of one with negative effects of the other.

Unexpectedly, in the path models (B), which used unreciprocated ties, the numbers of such ties were positively associated with both desire and depression symptoms, and thus these effects were opposite in sign depending on whether reciprocated or unreciprocated friendships were used; adolescents who made more unreciprocated friendship nominations were more likely to have a stronger desire to increase their number of friends, and to report more depression symptoms. Similarly, significant univariate regressions of reciprocated or unreciprocated friendships on desire and on social anxiety were also in opposite directions, such that decreasing number of reciprocated friendships or increasing number of unreciprocated friendships both predicted stronger desire and greater symptoms of social anxiety. While this confirms the prediction that the association with desire would be less negative for unreciprocated than reciprocated friendships, both associations were predicted to be negative. The unexpected finding that the association of unreciprocated friendships with desire was positive also conflicts with previous work suggesting that unreciprocated friendship nominations reflect a looser tie, but qualitatively similar effects (Lin & Weinberg, 2014). One possible explanation for this finding is that desire may reflect the perceived (in)adequacy of current ties, and reciprocated ties contribute to the perception of adequacy (thereby lowering desire) because of the social support offered to those perceived as friends. If so, unreciprocated ties may imply that the adolescent perceives as friends individuals who do not offer such support, which may lower the expected benefit of each additional friendship, and therefore increase the extent to which an increase in numbers of friends appears to be required for a given amount of benefit. Alternatively, the direction of causation may be inverted for unreciprocated nominations: desire may influence unreciprocated friendship nominations through a kind of “wishful thinking” wherein the desire for more friendships lowers the threshold when selecting who to nominate (see e.g., Scholte et al., 2009). Future studies of adolescent social networks examining the differences and similarities between reciprocated and unreciprocated ties would allow further interpretation of this finding.

### Limitations

This study employed a path model analysis of cross-sectional data. This statistical method allows one to perform regression analyses on a complex, multi-step equation, but is unable to distinguish between causal and merely correlational effects using cross-sectional data. A future study using a longitudinal design would provide more robust support for possible causal relations between these constructs. Furthermore, substantial evidence shows bidirectional effects between internalizing symptoms and social inclusion (e.g., Jacobson & Newman, 2016). For example, anxious withdrawal and social exclusion are mutually reinforcing (Gazelle & Ladd, 2003). Similarly, social anxiety entails information-processing biases that can hamper accurate detection of social threat (Blöte et al., 2010), and depression is associated with shyness (Murberg, 2009) and other difficulties in social interactions (Segrin & Flora, 2000; see also Hames, et al., 2013). A longitudinal (repeated measures) design would also permit future studies to differentiate between the effects on internalizing symptoms of; friendship networks, desire, and SGO; and the inverse effects. Furthermore, the use of a single item to measure desire prevents estimation of Chronbach’s alpha (the standard statistical estimate of the reliability of a psychometric, which assumes multiple items administered concurrently) and reduces construct validity since differences between participants’ interpretation of the item cannot be “averaged out” by summing multiple items. Moreover, while we treated the desire variable as ordinal, and not continuous, in recognition of the low sensitivity of a five-point single-item measure, the low sensitivity remains.

There are also limitations to our approach to estimating effects of friendships. Firstly, we estimated these effects based on number of reciprocated nominations alone. While the number of friends is of direct importance to the theorized relationships through desire, future studies could improve on our design by accounting for differences in the strength of friendships, as well as their duration (e.g., asking whether a friendship pre-dates the transition to secondary school), as a moderator or similar. Another limitation is that while self- and peer-report measures facilitate the collection of data from larger samples, the resulting data provide a less direct insight into how participants actually behave in social contexts than would be provided by directly observing them interacting. Furthermore, social goals and behavior may vary across different contexts (Erdley et al., 1997) whereas the peer nomination data used here were specific to the classroom, and so the measures of social relationships were focused on those peer relationships. However, in contrast with depressive symptoms’ negative association with number of friends at school, adolescents who report having more friends outside school also report more depressive symptoms (Ueno, 2005), suggesting friendships outside school cannot compensate for a lack of school friends. Additionally, parent-child relationships are also clearly important contributors to vulnerability or resilience to internalizing symptoms (Pössel et al., 2018). The present findings thus describe an observation about academically-able Dutch early adolescents in the specific context of classroom relationships with peers (see also; Henrich et al., 2010), which can be generalized to similar groups and, in concert with future work in other cultures and contexts and with different samples, may subsequently be found to generalize outside of western European classrooms.

### Directions for Future Research

As discussed above, future research should employ longitudinal designs and examine these dynamics also in other contexts (e.g., the family, or in another cultural context). Peer nomination studies comparing reciprocated and unreciprocated ties are also needed. Furthermore, responses to failures and successes have been studied in relation to goal orientation, with demonstration goals predicting faster withdrawal of effort and persistence (Sideridis & Kaplan, 2011), and increased helplessness (Erdley et al., 1997), and negative interpersonal experiences exaggerate the effects of social goals on adjustment (Kuroda & Sakurai, 2011). Future research should investigate the influence of experiences of social “failures” on the interplay between social goals, perceptions of status and closeness, self-disclosure and silencing, and adjustment. It may be important for studies of social “failure” experiences to examine the possibility of a division of the development goals concept into goals of increasing skill and goals of avoiding reducing skill (Lou, Masuda & Li, 2017; see also Larsen et al. (2012); and Pearson & Rose, 2021). Finally, the present results support the proposition that status-oriented goals would compete with closeness-oriented goals for priority in guiding behavior. Future research should investigate whether status-oriented goals reduce closeness-seeking behaviors such as self-disclosure. Indeed, self-silencing (the antonym of self-disclosure) in friendships has been found to mediate the deleterious effect of rejection sensitivity on self-reported feelings of friendship support (Thomas & Bowker, 2015).

## Conclusion

While the protective effects of friendships have been extensively studied in relation to adolescents’ vulnerability to internalizing symptoms, the possibility that qualitative differences in social motivation could play a role in producing or amplifying these effects has received little attention. The path models tested here rest on the hypothesis that a strong desire for more friends in the bounded community of a classroom would promote demonstration (and thus status) -oriented social goals, which would in turn promote internalizing symptoms. As predicted, the link between numbers of friendships and internalizing symptoms was mediated in two steps. First, adolescents with fewer reciprocated classmate friendships expressed a stronger desire for more classmate friendships. Second, adolescents who expressed a stronger desire for more friendships endorsed more demonstration-avoidance goals, which in turn predicted more internalizing symptoms. This may reflect a tendency toward status-oriented social behavior in adolescents with a strong desire for more friends, and increased attention to social status may come at the expense of cultivating interpersonal intimacy in extant friendships, and promote psychosocially maladaptive attention allocation in social situations. However, not all predictions were supported; no beneficial effect of development goals was observed, and number of unreciprocated friendship nominations was positively associated with desire for more friendships and social anxiety. Future research should seek to examine bidirectional or cyclical relationships in this domain using a longitudinal design.

### Supplementary Information

Supplementary Materials
