# Correction: A Few Close Friends? Adolescent Friendships’ Effect on Internalizing Symptoms Is Serially Mediated by Desire for More Friends and Social Goal Orientation

**DOI:** 10.1007/s10964-023-01889-1

**Published:** 2023-10-28

**Authors:** Reubs J. Walsh, Nikki C. Lee, Imke L. J. Lemmers-Jansen, Miriam Hollarek, Hester Sijtsma, Mariët van Buuren, Lydia Krabbendam

**Affiliations:** 1 Department of Clinical, Neuro-, and Developmental Psychology, Faculty of Behavior and Movement Sciences, Vrije Universiteit Amsterdam, Amsterdam, the Netherlands; 2 Institute for Brain and Behavior Amsterdam, Vrije Universiteit Amsterdam, Amsterdam, the Netherlands; 3 LEARN! Interfaculty Research Institute, Vrije Universiteit Amsterdam, Amsterdam, the Netherlands; 4 Department of Developmental Psychology, Utrecht University, Utrecht, the Netherlands; 5 Department of Psychosis Studies, Institute of Psychiatry, Psychology and Neuroscience, King’s College London, London, UK

Correction to: Journal of Youth and Adolescence (2013) 52:1-17 10.1007/s10964-023-01780-z

In the original publication of the article, Table 1 contained t-statistics where the authors intended to give correlation coefficients. The authors apologize for this error which has now been corrected.

**Table 1:** Means, standard deviations, and correlations for all model variables

	Mean	SD	1.	2.	3.	4.	5.	6.	7.	8.
1. Desire	2.118	1.162		0.174***	0.380***	0.172***	0.375***	0.328***	−0.309***	0.185***
2. Depression Symptoms	7.953	7.956	0.185***		0.502***	0.081	0.220***	0.138**	−0.112*	0.067
3. Social Anxiety Symptoms	38.612	11.81	0.380***	0.515***		0.197***	0.584***	0.303***	−0.237***	0.113*
4. Development	3.148	0.850	0.178***	0.109*	0.228***		0.312***	0.153**	−0.007	0.131*
5. Demonstration-Avoidance	2.990	0.810	0.365***	0.234***	0.594***	0.323***		0.408***	−0.190***	0.067
6. Demonstration-Approach	2.194	0.809	0.330***	0.151**	0.331***	0.174***	0.417***		−0.032	0.134*
7. Reciprocated Friendships	4.111	2.201	−0.331***	−0.107*	−0.253***	−0.004	−0.189***	−0.035		0.071
8. Unreciprocated Friendships	2.466	2.196	0.185***	0.080	0.103*	0.132*	0.065	0.131*	0.071	

Correlations between the untransformed variables are given above the diagonal, and their correlations after the square-root transformation are given below the diagonal

*p ≤ 0.05. **p ≤ 0.01. ***p ≤ 0.001

