# Supplementary material for: A Few Close Friends? Adolescent Friendships’ Effect on Internalizing Symptoms Is Serially Mediated by Desire for More Friends and Social Goal Orientation
Source: J Youth Adolesc. 2023 Apr 30;52(7):1357–73. doi: 10.1007/s10964-023-01780-z (PMC10175381; doi:10.1007/s10964-023-01780-z)
Supplement: Supplementary file 1 — Supplementary Materials [file 10964_2023_1780_MOESM1_ESM.docx]

**Supplementary Materials for:**

**A Few Close Friends? Adolescent Friendships’ Effect on Internalizing Symptoms Is Serially Mediated by Desire for More Friends and Social Goal Orientation**

Reubs J Walsh*^1,2,3^, Nikki C. Lee^1,2,3,4^, Imke L. J. Lemmers-Jansen^1,2,3,5^, Miriam Hollarek^1,2,3^, Hester Sijtsma^1,2,3^, Mariët van Buuren^1,2,3^, and Lydia Krabbendam^1,2,3^.

1. Clinical, Neuro-, and Developmental Psychology, Faculty of Behavior and Movement Sciences, Vrije Universiteit Amsterdam
2. Institute for Brain and Behavior Amsterdam, Vrije Universiteit Amsterdam, Amsterdam, the Netherlands
3. LEARN! Interfaculty Research Institute, Vrije Universiteit Amsterdam, Amsterdam, the Netherlands
4. Department of Developmental Psychology, Utrecht Universiteit, Utrecht, the Netherlands
5. Department of Psychosis Studies, Institute of Psychiatry, Psychology and Neuroscience, King’s College London, London, United Kingdom

*Corresponding Author: Reubs J Walsh, MF-5.38, Van der Boechorststraat 9, 1081 BT Amsterdam, The Netherlands. Tel: +31 20 59 89777. Email: r.j.walsh@vu.nl

**Submitted to: Journal of Youth and Adolescence.**

**Data Preprocessing**

***Peer Nomination Data Correction***

Data correction procedure for peer nomination data is based on a previously tested method (Velásquez, Bukowski, & Saldarriaga, 2013), in which a regression analysis was used to obtain an estimate of the (curvilinear) effect of class size on numbers of nominations made and received by participants. Due to a greater degree of variation in participation rates in our sample than reported in Velásquez et al (2013), we added participation rate as an additional confounder to regress out. Therefore we specified regression models of the general form

$$N \sim S + S^{2}+ i+ R + \text{ε}_{\text{c}}$$

where $N$ is the number of nominations, $S$ is class size, $R$ is the participation rate for the class (i.e. $R=P/S$, where $p$ is the number of participants in the class) and $i$ is an interaction term $S\times p$. The variables $N, S, R,$ and $P$ were standardised (i.e. z-scored) and $i$ was then calculated from these values. The random effect term $\text{ε}_{\text{c}}$ accounts for differences between the groups arising from factors other than size and participation rate. Step-wise removal of terms (model preference based on an improvement in the Bayesian Inference Criterion) yielded the following two equations:

$$N_{in}\sim S + R + \varepsilon_{c}$$

$$N_{out}\sim S + S^{2}+ R + \varepsilon_{c}$$

where $N_{in}$ is the total number of nominations the participant receives and $N_{out}$ is the total number of nominations made by the participant. The number of nominations were therefore corrected by calculating the predicted value of $N$, omitting the intercept and random effects:

$$P_{in}= \beta_{S}S + \beta_{R}R$$

$$P_{out}= \beta_{S}S + \beta_{S^{2}}S^{2}+ \beta_{R}R$$

where $P_{in}$ and $P_{out}$ are the predicted numbers of nominations received and made, respectively, based on group size and participation rate, and $\beta$'s are the corresponding regression estimates. These regressions were performed on the full data-set (including classes with below the 70% participation threshold).

The numbers of nominations received or made by an individual on a particular question was corrected thus:

$$Y^{'}= Y \frac{A - P}{A}$$

where $Y'$ is the corrected value, $Y$ is the raw number of nominations, and $A$ is the total number of nominations (either $in$ or $out$; likewise, $P$ is either $P_{in}$ or $P_{out}$, depending on which variable is being corrected).

Reciprocal ties were corrected using the formula:

$$Y^{'}= Y\cdot\frac{A_{in}- P_{in}}{A_{in}}\cdot\frac{A_{out}- P_{out}}{A_{out}}$$

thereby accounting for both the in-link and out-link aspect of the reciprocal tie (or in other words, the effect of participation rate on both the nomination *by* the participant, and the nomination *of* the participant).

***Data normalisation***

Prior to analysis, we examined the data with Q-Q plotting to identify violations of the assumption of normality. Each variable was significantly right-skewed and failed a Shapiro-Wilkes test of normality (*p*<0.05). To increase the normality of the data distributions, whilst ensuring that the linear relationships modelled were indeed modelled as linear, we applied the same transformation to every variable, which was to take the square-root of the Z-scored value. This produced a marked improvement in the Q-Q plots and we proceeded to the planned path model analysis. Note that the corrected estimate of unreciprocated (outlink) friendship nominations was in a small (n=4) number of cases a negative value. Therefore, on this variable we used the transformation $\sqrt{Y^{'}+1}.$ All subsequent analyses used these (corrected) transformed data.
